# Supplementary material for: Impact of Anti-SARS-CoV-2 Vaccination on Disease Severity and Clinical Outcomes of Individuals Hospitalized for COVID-19 Throughout Successive Pandemic Waves: Data from an Italian Reference Hospital
Source: Vaccines (Basel). 2024 Sep 6;12(9):1018. doi: 10.3390/vaccines12091018 (PMC11435849; doi:10.3390/vaccines12091018)
Supplement: Supplementary file 1 [file vaccines-12-01018-s001.zip › vaccines-3166172-supplementary.pdf]

SUPPLEMENTARY MATERIALS

Impact of anti-SARS-CoV-2 vaccination on disease severity and clinical outcomes of individuals hospitalized for COVID-19 throughout successive pandemic waves: data from an Italian reference hospital.

Authors:

Annalisa Mondi, Ilaria Mastrorosa, Assunta Navarra, Claudia Cimaglia, Carmela Pinnetti, Valentina Mazzotta, Alessandro Agresta, Angela Corpolongo, Alberto Zolezzi, Samir Al Moghazi, Laura Loiacono, Maria Grazia Bocci, Giulia Matusali, Alberto D'Annunzio, Paola Gallì, Fabrizio Maggi, Francesco Vairo, Enrico Girardi, Andrea Antinori.

Contents of Supplementary Materials

Supplementary methods, page 3

I. Classification of comorbidities

Supplementary results, page 4

I. Figure S1, page 4

II. Table S1a-b, page 5

III. Table S2a-b, page 6

Classification of comorbidities

- Diabetes
- Cardiovascular disease (any disease involving heart or blood vessels, such as coronary artery diseases, heart failure, hypertensive heart disease, rheumatic heart disease, cardiomyopathy, arrhythmias, valvulopathies, congenital heart disease, peripheral artery disease)
- Chronic respiratory disease (such as chronic obstructive pulmonary disease, asthma, Interstitial lung disease; tuberculosis is also included)
- Metabolic disease (obesity and dyslipidemia)
- Renal disease (any chronic kidney disease, such as glomerular diseases, inherited conditions, autoimmune conditions, including participants under dialysis)
- Neoplasms/hematologic diseases (any active malignant solid neoplasm or onco-hematologic disease such as leukemia, lymphoma, multiple myeloma; previous neoplasms or other hematologic diseases such as myelodysplastic syndrome are not included)
- Liver disease (chronic viral and non-viral hepatitis, compensated/decompensated cirrhosis)
- Immunodeficiency diseases (primary and secondary immunodeficiency disorders; participants with HIV or transplant recipients are also included).

Figure S1. Anti-SARS-CoV-2 vaccination coverage in Lazio Region (red) and in the study population (black), including only individuals hospitalized for COVID-19, represented for each trimester of the study period (January 2021-June 2023), both overall and by age group.

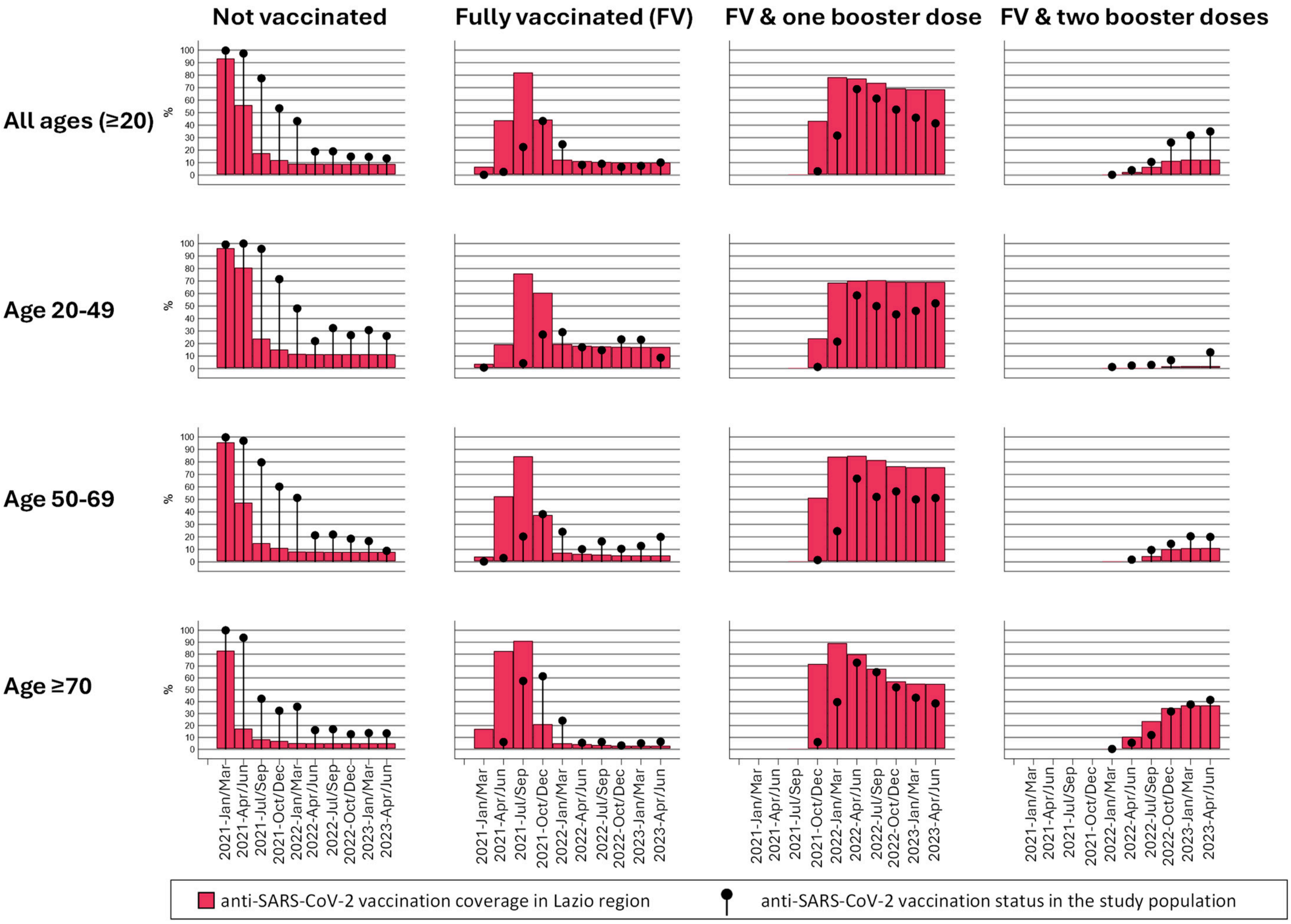

Table S1. Univariable logistic regression analysis for a) in-hospital death and b) clinical progression within 28 days from hospital admission, in the entire study population (n=4,488).

Characteristics	a) 28 days-Death			b) 28 days-Clinical progression		
	OR	95% CI	p-value	OR	95% CI	p-value
**Vaccination status**						
Not vaccinated	1.00			1.00		
Fully vaccinated	1.12	0.83–1.52	0.450	0.83	0.66–1.05	0.116
Fully vaccinated and one booster dose	1.17	0.91–1.49	0.214	0.75	0.61–0.91	0.003
Fully vaccinated and two booster doses	1.12	0.76–1.63	0.573	0.75	0.56–1.02	0.069
**Sex**						
Male	1.00			1.00		
Female	0.76	0.62–0.95	0.014	0.70	0.60–0.83	<0.001
**Age** (for 10 years increase)	1.55	1.44–1.67	<0.001	1.20	1.14–1.26	<0.001
**Country of birth**						
Italy	1.00			1.00		
Abroad	0.49	0.32–0.74	0.001	0.53	0.40–0.72	<0.001
**Comorbidities (Type)**						
Diabetes	1.39	1.07–1.80	0.013	1.32	1.08–1.62	0.008
Cardiovascular disease	2.43	1.95–3.04	<0.001	2.10	1.78–2.48	<0.001
Chronic respiratory disease	1.34	1.06–1.70	0.016	1.22	1.01–1.47	0.041
Metabolic disease	1.31	0.95–1.81	0.103	1.56	1.23–1.99	<0.001
Renal disease	4.07	3.10–5.34	<0.001	2.67	2.09–3.40	<0.001
Neoplasms/hematologic diseases	2.80	2.07–3.78	<0.001	1.75	1.34–2.30	<0.001
Liver disease	0.68	0.35–1.29	0.237	0.67	0.41–1.09	0.109
Immunodeficiency diseases	1.31	0.79–2.16	0.297	0.84	0.53–1.31	0.437
**Previous infection**						
No	1.00			1.14	0.92–1.41	0.246
Yes	0.65	0.38–1.11	0.112	1.00		
**Pandemic wave**						
Alpha (≤18/07/2021)	0.64	0.48–0.84	0.001	1.08	0.89–1.31	0.431
Delta (19/07/2021–20/12/2021)	0.78	0.59–1.03	0.076	1.17	0.96-1.43	0.129
Omicron (≥21/12/2021)	1.00			1.00		

Abbreviations: n, number of participants; IQR, interquartile range; OR, odds ratio; CI, confidence interval.

Table S2. Univariable logistic regression analysis for a) in-hospital death and b) clinical progression within 28 days from hospital admission, restricted to vaccinated study participants (n=2,726).

Characteristics	a) 28 days-Death			b) 28 days-Clinical progression		
	OR	95% CI	p-value	OR	95% CI	p-value
**Vaccination status**						
Fully vaccinated	1.00			1.00		
Fully vaccinated and one booster dose	1.04	0.75–1.44	0.815	0.90	0.69–1.17	0.436
Fully vaccinated and two booster doses	0.99	0.64–1.53	0.974	0.91	0.64–1.29	0.595
**Time elapsed from the last dose**						
≤120 days	1.00			1.00		
>120 days	1.59	1.06–2.38	0.026	1.04	0.77–1.40	0.802
**Sex**						
Male	1.00			1.00		
Female	0.70	0.52–0.94	0.019	0.67	0.53–0.86	0.001
**Age** (for 10 years increase)	1.44	1.28–1.62	<0.001	1.18	1.09–1.28	<0.001
**Country of birth**						
Italy	1.00			1.00		
Abroad	0.51	0.25–1.05	0.068	0.56	0.32–0.98	0.043
**Comorbidities (Type)**						
Diabetes	1.36	0.97–1.90	0.074	1.46	1.11–1.93	0.007
Cardiovascular disease	1.93	1.41–2.63	<0.001	1.70	1.32–2.18	<0.001
Chronic respiratory disease	1.05	0.76–1.45	0.761	1.27	0.98–1.64	0.065
Metabolic disease	0.95	0.55–1.61	0.839	1.04	0.68–1.59	0.869
Renal disease	3.30	2.34–4.67	<0.001	2.62	1.92–3.57	<0.001
Neoplasms/hematologic diseases	2.85	2.00–4.05	<0.001	2.08	1.52–2.86	<0.001
Liver disease	0.53	0.21–1.31	0.168	0.67	0.35–1.31	0.247
Immunodeficiency diseases	1.25	0.69–2.27	0.464	1.08	0.65–1.82	0.759
**Previous infection**						
No	1.00			1.00		
Yes	0.74	0.41–1.36	0.340	0.61	0.36–1.03	0.064
**Pandemic wave**						
Alpha (≤18/07/2021)	0.58	0.08–4.41	0.601	0.77	0.17–3.38	0.728
Delta (19/07/2021–20/12/2021)	0.77	0.50–1.19	0.240	0.95	0.68–1.32	0.766
Omicron (≥21/12/2021)	1.00			1.00		

Abbreviations: n, number of participants; IQR, interquartile range; OR, odds ratio; CI, confidence interval.
